# The rat retrosplenial cortex is required when visual cues are used flexibly to determine location^☆^

**DOI:** 10.1016/j.bbr.2014.01.028

**Published:** 2014-04-15

**Authors:** E.L. Hindley, A.J.D. Nelson, J.P. Aggleton, S.D. Vann

**Affiliations:** School of Psychology, Cardiff University, Park Place, Cardiff, Wales CF10 3AT, UK

**Keywords:** Cingulate cortex, Spatial memory, Orientation, Perspective, Navigation

## Abstract

The present study examined the consequences of retrosplenial cortex lesions in rats on two novel spatial tasks. In the first experiment, rats discriminated opposing room views from the same general location, along with their opposing directions of travel (‘Perspective’ task). Rats were trained with food rewards using a go/no-go design. Extensive retrosplenial cortex lesions involving both the granular and dysgranular areas impaired acquisition of this discrimination, which relied on distal visual cues. The same rats were then trained on a non-spatial go/no-go discrimination between different digging media. No lesion effect was apparent. In the final experiment, rats discriminated between two locations within a room (‘Location’ task) such that direction of travel at each location would be of less help in solving the problem. Both extensive retrosplenial lesions and selective dysgranular retrosplenial lesions impaired this Location task. These results highlight the importance of the retrosplenial cortex (areas 29 and 30), including the dysgranular cortex (area 30), for the effective use of distal visual cues to solve spatial problems. The findings, which help to explain the bias away from visual allocentric solutions that is shown by rats with retrosplenial cortex lesions when performing spatial tasks, also support the notion that the region assists the integration of different categories of visuospatial information.

## Highlights

• Combined loss of areas 29 and 30 impairs place discriminations.
• Area 30 is needed for the control of place learning by visual cues.
• Novel discrimination tasks described for place and perspective learning.
• Areas 29 and 30 assist the integration of different classes of spatial information.

## 1 Introduction

Multiple areas in the rodent brain appear to support spatial learning and navigation [1]. The assumption is that these multiple areas function in different but complementary ways. The retrosplenial cortex (areas 29 and 30) is one such site. Both its connectivity and the outcome of lesion studies strongly suggest that the spatial functions of the retrosplenial cortex region are closely linked to those of the hippocampus and the anterior thalamic nuclei [2–9]. Consistent with this notion is the finding of head direction cells in the rat retrosplenial cortex [10,11]. These neurons signal the direction an animal is heading, independent of location. Additional support comes from studies of patients with pathologies involving the retrosplenial cortex, particularly in the right hemisphere [12,13]. Many of these patients report an inability to use familiar visual landmarks to navigate, despite retaining knowledge of the landmarks themselves, while some appear unable to orient themselves either in a novel or familiar environment [12,13]. A barrier to determining the particular importance of the human retrosplenial cortex is the lack of patients with pathologies confined to this region [14].

To address this issue, selective retrosplenial cortex lesions have been examined in rats. Previous studies have found spatial deficits in a variety of behavioural protocols, including water-maze, T-maze and radial-arm maze tasks [7,15–19]. These same lesion studies often reveal a reluctance by the rats to use allocentric visual cues when other spatial strategies are available. In addition, spatial tests in the dark have implicated the retrosplenial cortex in some forms of idiothetic learning [19–21], leading to the notion that this region may assist in the integration of visual with idiothetic spatial information [22]. A related, broader notion is that the retrosplenial cortex has a ‘translational’ function in the integration and transformation between multiple spatial codes, including allocentric representations into egocentric ones and *vice versa* [14,23,24]. Support comes from fMRI studies showing that the retrosplenial cortex is activated during a task requiring people to imagine looking at the same scene from different viewpoints [25,26].

The present study sought to examine the effects of retrosplenial cortex lesions on two related spatial tasks where the use of distal visual cues could be assessed. The first task concerned the ability to distinguish the features of a room from a set viewpoint (‘Perspective’ task, Experiment 1). The second task concerned the ability to discriminate between two locations within a room, irrespective of the direction faced (‘Location’ task, Experiment 3). These closely related tasks were selected as the demands of the second task build onto those of the first task, including the ability to unite different perspectives from the same place.

Two cohorts of rats were examined, the first with lesions in both areas 29 and 30, the second with lesions targeted at just area 30 (dysgranular retrosplenial cortex). The goal was to test whether the dysgranular cortex is a critical access point for visual processing within the retrosplenial region. However, only the findings from the first cohort (combined area 29 and 30 lesions) are described for the ‘Perspective’ task (Experiment 1). This omission reflects procedural differences that restrict comparisons across the two cohorts (see below). Both Experiments 1 and 3 used go/no-go discriminations where rats were only reinforced for digging when in the correct viewpoint or location. Experiment 2, therefore, tested whether such go/no-go procedures, with their emphasis on withholding responses, are appropriate for rats with retrosplenial damage. In this control study, rats were trained on a non-spatial go/no-go task involving the discrimination of different cups containing distinct digging media.

## 2 Methods

### 2.1 Experimental methods

Two cohorts of animals were trained and tested. Both cohorts completed all three experiments in the same rooms. For Experiment 1 there were, however, procedural differences in the number and type of probe tests, the use of background white noise, and the immediately prior behavioural task. The control rats in Cohort 1 acquired the Perspective task at about twice the rate of their counterparts in Cohort 2, further weakening any cohort comparisons. For these reasons, the methods and results from Cohort 2 are not described for Experiment 1. Thereafter, the training of the two Cohorts was matched.

#### 2.1.1 Animals

Subjects were 52 male Lister Hooded rats (Harlan, Bicester, UK). The rats were housed in pairs in a temperature-controlled room. Lighting was kept on a 12-hour light/dark cycle, from 08:00 to 20:00. Water was available *ad libitum* throughout the experiments. For all behavioural experiments, the animals were placed on a food-restricted diet where they were able to gain weight. Their weights did not fall below 85% of their free-feeding weights. All experiments were carried out in accordance with UK Animals (Scientific Procedures) Act, 1986 and associated guidelines, and were approved by local ethical committees (Cardiff University). Rats were provided with cardboard tubes and wooden chew sticks in their home cages. Animals in Cohort 1 received either a bilateral combined excitotoxic lesion of areas 29 and 30 (RScomb, *n* = 16) or a sham lesion (Sham1, *n* = 12). Their weight at the time of surgery was between 278 and 387 g. Subjects for the dysgranular cortex study (Cohort 2, 294–314 g) received either a bilateral excitotoxic lesion within area 30 of the retrosplenial cortex (RSdysg, *n* = 14) or a sham lesion (Sham2, *n* = 10).

#### 2.1.2 Surgical procedures

Rats were deeply anaesthetized with an intraperitoneal (i.p.) injection of sodium pentobarbital (60 mg/kg pentobarbital sodium salt; Sigma-Aldrich, U.K.). All animals were given a subcutaneous injection of 0.06 ml Metacam (Boehringer Ingelheim, Alkmaar, NL, USA) to reduce post-operative pain, as well as 0.1 ml Millophylline (i.p.; Arnolds Veterinary Products Ltd, Shrewsbury, UK) to regulate breathing. The scalp was shaved and the animal then placed in a stereotaxic frame (David Kopf Instruments, Tujunga, CA, USA) with the nose bar set at +0.5. The skull was exposed and a bilateral craniotomy extending from bregma to lambda was made in the skull using a dental drill. Consequently, two strips of skull above the retrosplenial cortex were removed, leaving a bridge of bone approximately 2 mm wide along the length of the central sinus.

Lesions were made by injecting 0.09 M N-methyl-D-aspartate (NMDA; Sigma, Poole, UK) dissolved in phosphate buffer (pH 7.2), into 14 injection sites at a rate of 0.05 μl per minute using a 1 μl Hamilton syringe (gauge 25s; Bonaduz, Switzerland). The stereotaxic coordinates of the lesion placements are stated relative to bregma in the anterior-posterior (AP) axis, and relative to the central sinus in the lateral-medial (LM) axis. Dorsal-ventral (DV) coordinates are taken relative to the surface of the cortex, using the eye of the needle.

The coordinates for the combined lesion cohort (RScomb) were AP-1.6, LM ± 0.4, DV-1.3; AP-2.8, LM ± 0.5, DV-1.4; AP-4.0, LM ± 0.5, DV-1.4; AP-5.3, LM ± 0.5, DV-2.6; AP-5.3, LM ± 0.9, DV-1.6; AP-6.6, LM ± 1.0, DV-2.0; AP-7.5, LM ± 1.1, DV-1.3. Injections of 0.25 μl NMDA were made in the three most rostral pairs of sites, with 0.26 μl injected in the next three pairs of sites. In the most caudal site 0.1 μl was injected. Coordinates for the dysgranular lesion group (RSdysg) were: AP -1.6, LM ± 0.4, DV-1.0; AP-2.8, LM ± 0.5, DV-1.1; AP-4.0, LM ± 0.5, DV-1.1; AP-5.3, LM ± 0.5, DV-2.4; AP-5.3, LM ± 0.9, DV-1.4; AP-6.6, LM ± 0.9, DV-1.8; AP-7.5, LM ± 1.0, DV-1.1. At each site 0.25 μl of NMDA was injected, apart from for the most caudal pair of injections, where the injections were 0.1 μl NMDA.

After each infusion the needle was left in place for 5 min before being slowly withdrawn. On occasion, animals received an additional dose of 0.05 ml sodium pentobarbital to maintain anaesthesia. If further anaesthesia was still required <2% inhaled isoflurane was given. Oxygen was provided throughout the surgery. Following surgery, the scalp was sutured and a subcutaneous injection of 5 ml glucose-saline was given to replace lost fluids. Lidocaine (Xylocaine, AstraZeneca, UK) and antibiotic powder (Dalacin C, Pharmacia, UK) were applied topically to the wound and animals were left to recover in a warm, quiet area before being returned to their home cage. Sham animals underwent the same procedure, except that the needle was not lowered and injections of neurotoxin were not made. Post-operative care was identical for all groups. All animals recovered well following surgery.

### 2.2 Experiment 1 - viewpoint discrimination (‘perspective’ task)

Only the procedure for Cohort 1 is described.

#### 2.2.1 Pre-training

Pre-training for the combined retrosplenial lesion rats (Cohort 1) started two months after surgery and followed testing on an object-in-place task. All rooms used in the study were novel to the rats. During pre-training, two round digging cups (6.5 cm tall and 7 cm in diameter) made of black plastic were used to train rats to dig for food rewards. A false bottom, made of metal grille, was inserted into the base to allow Cheerios (Nestle, UK) to be hidden beneath where they could be smelled by the rats but not accessed. The cups could be fixed with Velcro to the floor of the pre-training arena, a rectangular white plastic box measuring 25 cm × 42 cm with walls 12 cm high. The cups were placed 2 cm away from the short end wall of the arena. Habituation took place in a room measuring 195 cm × 330 cm with walls 255 cm high. This room was different to that used for subsequent testing.

The RScomb and Sham1 rats were habituated to the digging cups and arena over four days, receiving 10 min in the apparatus each day. The cups were filled with shredded paper mixed with crumbled Cheerios to mask the smell of a hidden Cheerio reward. One cup was placed at either end of the habituation arena. On the first day of habituation, half a Cheerio was placed on top of the paper in either cup. To encourage exploration of both cups, each cup was only re-baited after the other Cheerio had been eaten. Over the four sessions, the Cheerio was gradually buried deeper into the paper so that by the end of pre-training the rat would reliably dig to recover the food at the bottom of the digging cup. White noise at approximately 75 db was present throughout habituation. The source of the white noise was directly underneath the digging arena.

#### 2.2.2 Test procedure

##### 2.2.2.1 Apparatus

**Fig. 1:**
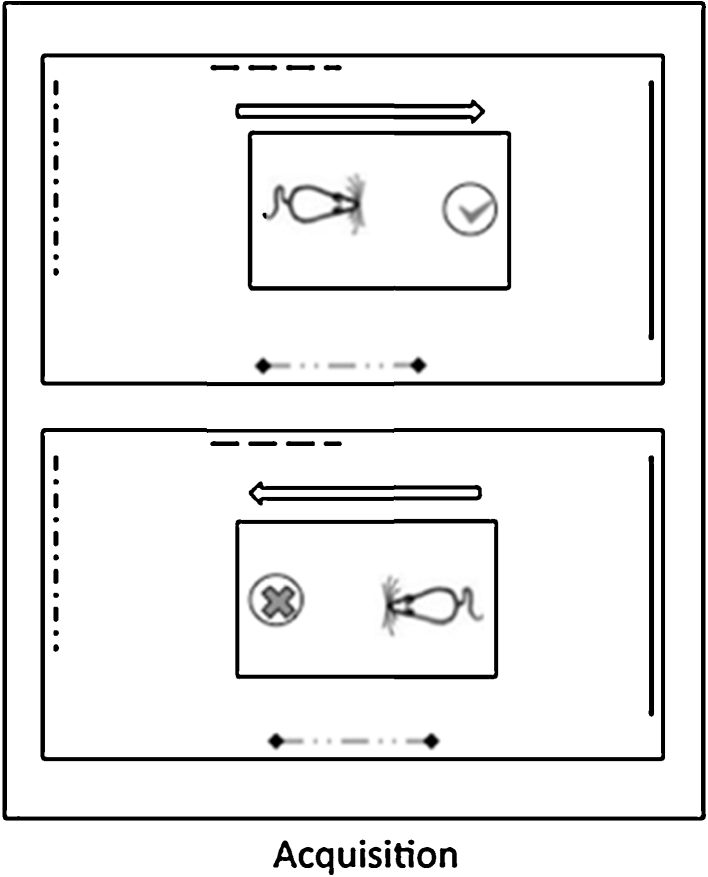
Perspective task (Experiment 1). A schematic of the two trial types in the Perspective task, which was used to test viewpoint discrimination. Rats were rewarded (tick) when the cup was in one direction relative to the animal's starting position (upper panel), but not rewarded (cross) if the rat had to travel in the opposite direction to reach the cup (lower panel). Visual cues (various dashed lines) were attached to the walls to help them to be distinguished. (The apparatus, room and test arena are not to scale).

The same digging cup was used for the pre-training and test phases. A larger test arena was used. The rectangular arena consisted of a clear plastic box (35 cm × 55 cm with walls 17 cm high). The digging cup was placed 2 cm from the middle of the short wall of the plastic box (Fig. 1). The arena was placed on a table, 70 cm high, in the centre of a room that measured 330 cm × 250 cm with walls 255 cm high. There were salient visual cues on the walls, such as posters and shapes made from coloured paper. The holding boxes were kept on two tables (76 cm high) that were located against the two walls of the room that the rats did not face during testing. For test trials in the light, the light level in the centre of the arena was 400 lux; for test trials in the dark the illumination was less than 1 lux.

##### 2.2.2.2 Behavioural protocol

During testing the rats were rewarded when the cup was in one direction relative to the animal's starting position e.g., East, but not if the rat had to travel in the opposite direction to reach the cup (e.g., West; see Fig. 1). The rewarded direction was counterbalanced across groups, as was the direction from which the rat was placed in the testing arena. Rats were carried to the test room in individual aluminium holding boxes, each with a hinged lid that prevented the rats from seeing the room when being brought in and when waiting between trials. Rats were tested in groups of four, each receiving one trial in turn. After initial training, the inter-trial interval was approximately 90s. Between trials the holding box was rotated to deter the rats from building up internal direction cues that could allow them to solve the task in a non-visual manner. In addition, the entire test arena was rotated 180° between each trial to avoid directional odour cues developing. The tester also varied their position with respect to the arena. The arena and cup were cleaned with alcohol wipes between each group of four rats.

On the first two days of training, each rat was placed in the test arena for 30s before the first trial. This additional period was to habituate the rats to the new arena. Every rat completed 16 trials per day, each trial lasting a maximum of 20s. Digging was defined as breaking the surface of the paper with the nose or paws. If the rat failed to dig on a correct (go) trial, the experimenter showed the rat the Cheerio at the end of the 20s period, and was allowed to eat the reward. If the rat successfully found the Cheerio it was given 5s to eat the reward before being returned to the holding box. On any incorrect trial the rat was removed from the box after 20s or after finishing digging, whichever came first. The latency to dig was recorded for each trial and a difference score calculated, i.e., dig latency on non-rewarded (no-go) trials minus the dig latency on rewarded (go) trials. A positive score was, therefore, evidence of successful discrimination between the non-rewarded and rewarded pots. A score of zero would indicate no discrimination. The final difference score used was the cumulative score across all of the trials.

After 28 days of testing, when both groups were well above chance, two probe conditions were introduced. Each probe consisted of two sessions. For the first probe a plain white curtain was closed around the testing arena, to block distal visual cues on the far walls (‘Curtain’ probe). The room was rectangular in shape and the curtain followed the walls so that the general shape of the room and the positions of the two tables were still visible. In the second probe, the test sessions were run with both the curtain drawn closed and the room lights switched off in order to remove all visual cues (‘Dark’ probe). Between each probe session the RScomb and Sham1 rats were retrained for one session on the original task, i.e., with all cue types available. The intention was to ensure that performance on the probe session was not unduly affected by extinction.

### 2.3 Experiment 2—Digging cup discrimination (non-spatial go/no-go)

Both Cohorts 1 and 2 were trained on this task using the same methods. For Cohort 1, training began 4 months after surgery, while for Cohort 2 the interval was 10 months after surgery. Testing began shortly after completion of Experiment 1.

#### 2.3.1 Apparatus

Two different digging cups were used. One cup was the same as used in Experiment 1, and the other was a square cup made of white plastic, measuring 8 cm × 8 cm × 6 cm. A metal grille was inserted into the base of both cups to allow Cheerios to be hidden inside without being accessible to the rats. The square cup was filled with small plastic beads (Hama beads, Malta Haaning Plastic A/S, Denmark) and the round cup with blue craft sand. The cup was fixed with Velcro onto the floor of the test arena, a rectangular white plastic box measuring 25 cm × 42 cm with walls 12 cm high. The cup was placed 2 cm away from one end of the arena, and was changed each time the digging medium changed. The arena was placed on a table (height 76 cm), which was close to a wall in a room measuring 195 cm × 330 cm. The same room was used for both training and habituation, with white noise (75 db) played throughout for both the RSdysg and RScomb cohorts.

#### 2.3.2 Behavioural protocol

Rats were presented with a digging cup filled with either craft sand or Hama beads, and were rewarded for digging in one medium but not the other. The rewarded medium was counterbalanced across groups. No habituation was given, as the experiment took place directly after Experiment 1. Rats were placed in the arena at the opposite end to the digging cup, and the time taken to start digging measured. During a rewarded (go) trial, rats that did not dig were shown the Cheerio. Rats were removed from the arena 5s after finding the reward. During no-go trials, the rat was removed from the arena after 20s if it had not dug or was removed after it had finished digging in the incorrect cup and found no reward. Digging was defined as breaking the surface of the digging medium with the nose or paws. Rats were given 16 spaced trials per day in groups of four animals, giving an inter-trial interval of approximately 100s. The arena and cups were cleaned with alcohol wipes after each session to help remove odour cues. A difference score was calculated in the same way as for Experiment 1.

### 2.4 Experiment 3—Place discrimination (‘location’ task)

The same behavioural procedures were used for the two cohorts.

#### 2.4.1 Apparatus

The digging cups matched those used as in Experiment 1, while the test arena was the same as in Experiment 2. The testing room measured 300 cm × 280 cm, with a ceiling height of 240 cm. Visual cues such as posters and shapes made of coloured paper were fixed to the walls of the testing room to differentiate between the different areas. Two identical tables, 70 cm high, were placed in diagonally opposite corners of the room. The test arena was placed on one of these tables for each trial. The digging cup was filled with shredded paper. Light levels were at 260 lux in both test arena locations. Rats were brought to the testing room in individual metal holding boxes with lids. Background white noise was played throughout training (see Experiment 1). During testing, the holding box sat on a Table 76 cm high, placed in the centre of the room.

#### 2.4.2 Behavioural protocol

**Fig. 2:**
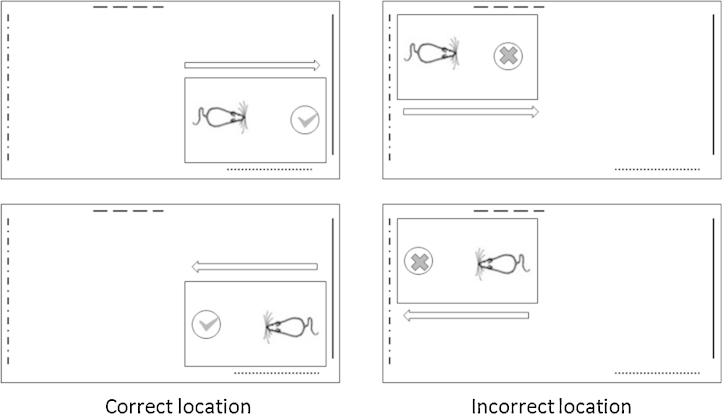
Location task (Experiment 3). In the Location task animals were rewarded for digging when the arena was in one corner of the room, but not if the arena was in the opposite corner. The animal could not use room direction *per se* to solve the task, as both directions were equally rewarded and unrewarded. Various visual cues (dashed lines) were attached to the walls to allow them to be distinguished. (The apparatus, room and test arena are not to scale).

This experiment required rats to discriminate between two distinct locations (Fig. 2). As this experiment directly followed Experiment 2, no habituation took place. There was only one test arena, which was moved between two identical tables in diagonally opposite corners of the room. Using the same arena throughout reduced the likelihood of using any local odour cues to discriminate location. To reduce odour cues further, the arena was cleaned with alcohol wipes after each session (the completion of a day's testing for four rats whose trials are interleaved with each other). Rats were rewarded for digging when in one arena location but not in the other, with the correct location counterbalanced across groups. Rats were placed in the arena so that they had to approach the cup from one of two directions in each location (see Fig. 2). As a consequence, direction *per se* was not a discriminative cue.

The procedure matched Experiment 1 in that rats were trained in groups of four, with an inter-trial interval of approximately 100 s. Each rat received 16 trials a day with a maximum of 20 s per trial. Following training, the RScomb and Sham1 rats were given a probe test in the dark, with light levels below 1 lux, to determine whether visual cues were critical for solving the discrimination. (Cohort 2 was not given this final probe.) All other running procedures were as previously described for Experiments 1 and 2.

### 2.5 Histological procedures

At the completion of all three experiments, rats were deeply anaesthetised using sodium pentobarbital (60 mg/kg, i.p.; Euthatal; Merial Animal Health, Harlow, UK), then transcardially perfused with 0.1 M phosphate-buffered saline (PBS) followed by 4% paraformaldehyde in 0.1 M PBS (PFA). The brains were removed and placed in PFA for 4 h before being transferred to 25% sucrose and left overnight at room temperature, with gentle agitation. Four adjacent series of coronal sections (40 μm) were cut on a freezing sliding microtome.

One series was mounted directly onto gelatin-coated slides after slicing and stained using cresyl violet, a Nissl stain. A second series was stained for NeuN, which is a selective marker for neurons that helps visualize the extent of any lesion. This second series was collected in PBS. To visualize NeuN, the free-floating sections were rinsed in 0.1 M PBST (PBS with 0.2% Triton X-100) and treated with 0.3% H_2_O_2_ (hydrogen peroxide) in 0.1 M PBST for 3 min to suppress endogenous peroxidase activity. Sections were rinsed four times in 0.1 M PBST for 10 min each time, and then incubated for 48 h at 4 °C in the monoclonal anti-NeuN serum (1:5000; Chemican, Temecula, CA, USA) diluted in PBST. After rinsing four times in 0.1 M PBST for a further 10 min each time, sections were incubated for 2 h in the secondary antibody, avidin-biotin-horseradish peroxidase complex (1:200; ABC-Elite, Vector Laboratories, Orton Southgate, Peterborough, UK) in PBST. After four rinses in 0.1 M PBST and two rinses in 0.05 M Tris buffer, sections were left for 1–2 min in a chromagen solution consisting of 0.05% diaminobenzidine (Sigma; Poole, UK), buffer solution and 0.01% H_2_O_2_ (DAB substrate kit; Vector Laboratories). The reaction was monitored visually and stopped by rinsing in cold 0.1 M PBS. The sections were mounted and dried on gelatin-coated slides. All slides (Nissl and NeuN) were then dehydrated through an alcohol series, cleared with xylene and cover-slipped using the mounting medium DPX.

### 2.6 Statistical methods

Statistical tests were carried out using SPSS 16.0 (SPSS Inc., Chicago). Where the assumption of sphericity was not met for parametric analysis, Greenhouse-Geisser corrections have been applied. In all statistical tests the critical alpha level is taken as *p* ≤ 0.05.

## 3 Results

### 3.1 Histological evaluation of the lesions

**Fig. 3:**
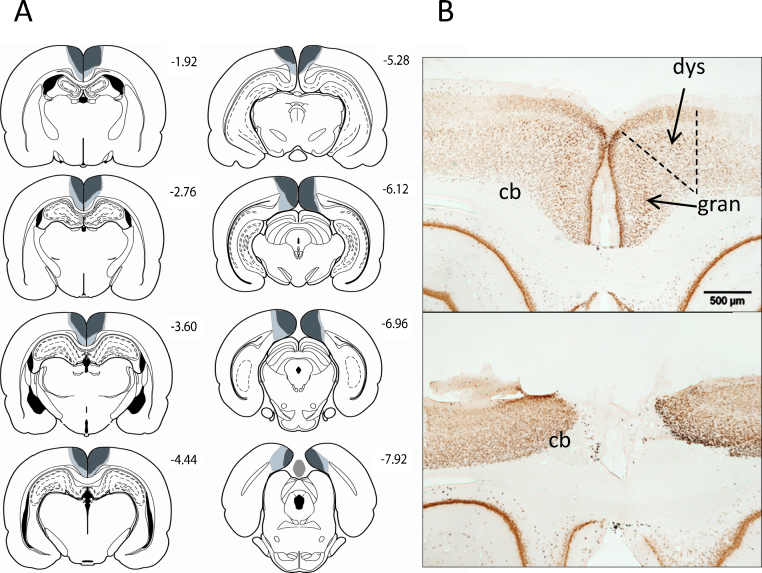
(A) A series of coronal sections showing the cases with the largest and smallest lesions included in the combined dysgranular and granular retrosplenial lesion group (RScomb). Light grey represents the largest lesion, and dark grey the smallest. The numbers correspond to the distance behind bregma in mm [50]. (B) Coronal NeuN sections showing the retrosplenial cortex (both hemispheres) in a sham surgery control rat (top), and a representative rat from the combined dysgranular and granular (RScomb) lesion group. The dashed lines show the limits of the retrosplenial cortex along with its granular and dysgranular sub-regions. The scale bar is 500 μm long. Abbreviations: cb, cingulum bundle; dys, dysgranular retrosplenial cortex; gran, granular retrosplenial cortex.

In the combined retrosplenial lesion cohort (RScomb) seven rats were excluded due to sparing of the retrosplenial cortex or due to bilateral damage to the hippocampus, leaving nine rats in the RScomb group and twelve corresponding controls (Sham1) (see Fig. 3). In these nine RScomb rats, extensive cell loss and gliosis was present throughout the retrosplenial cortex (Fig. 3) in both the granular and dysgranular sub-regions. Three of the nine animals had a very restricted area of cell loss and gliosis in the most dorsal medial tip of the CA1 subfield of the septal hippocampus. In two of these cases the CA1 cell loss was only unilateral. In one of these cases there was also some unilateral damage in the most medial part of the septal CA3. In all cases the CA damage was restricted to less than 600 μm in the AP direction. Four animals had partial sparing of the retrosplenial granular a subarea, particularly towards the caudal extent of the retrosplenial cortex. Three rats also had some limited sparing of retrosplenial granular b, typically only near its caudal limits. Only one animal showed a clear extension of the lesion into adjacent cortical areas, with some slight bilateral damage to the most caudal extent of the anterior cingulate cortex. A restricted area of gliosis was observed at the junction of the anterior medial and anterior ventral nuclei, as is consistently observed after extensive retrosplenial lesions [27–29]. No gliosis was seen in this area in the RSdysg animals.

**Fig. 4:**
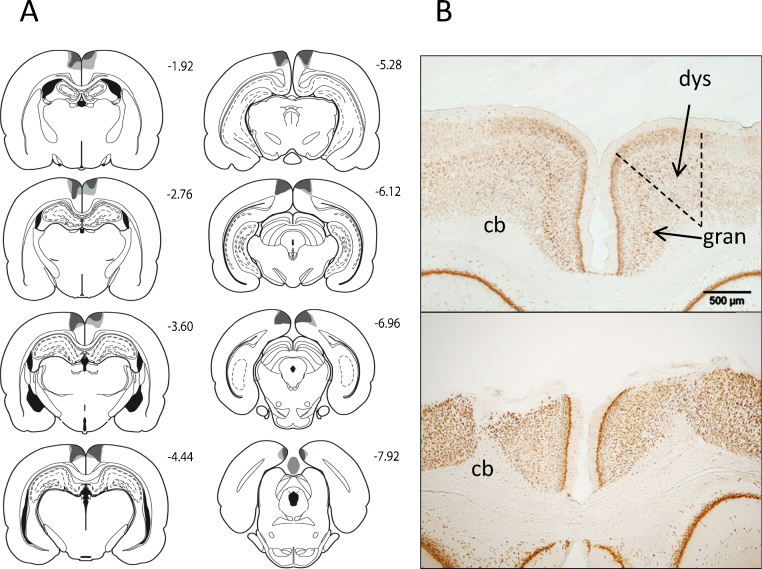
(A) A series of coronal sections showing the cases with the largest and smallest lesions included in the dysgranular retrosplenial lesion group (RSdysg). Light grey represents the largest lesion, and dark grey the smallest. The numbers correspond to the distance behind bregma in mm [50]. (B) Coronal NeuN sections showing the retrosplenial cortex (both hemispheres) in a sham surgery control rat (top) and a representative rat from the dysgranular (RSdysg) lesion group. The dashed lines show the limits of the retrosplenial cortex along with its granular and dysgranular sub-regions. The scale bar is 500 μm long. Abbreviations: cb, cingulum bundle; dys, dysgranular retrosplenial cortex; gran, granular retrosplenial cortex.

Six rats in the dysgranular retrosplenial lesion group (RSdysg) were excluded as the lesions were either largely unilateral or because there was a high level of bilateral sparing of the dysgranular retrosplenial cortex. The final number of subjects in the RSdysg group was eight, with ten in the corresponding sham group (Sham2). None of the remaining animals had damage to the hippocampus or the subiculum (Fig. 4). Five of the eight RSdysg animals had a very limited amount of unilateral damage to the granular retrosplenial cortex. The lesions did not extend into any other adjacent cortical areas (Fig. 4).

### 3.2 Experiment 1—viewpoint discrimination (perspective task)

Analyses of the digging latencies of Cohort 1 on the separate go and no-go trials over the first block of four sessions (prior to any evident learning) showed no differences in the initial dig latencies of the RScomb and Sham1 groups (*F* < 1). As the baseline latencies of the two groups appeared comparable, all further analyses focused on the difference in the latencies between the go and no-go trials. With task acquisition, this latency difference should increase. For each animal the latency difference between go and no-go trials was summed over the 16 trials in each session.

**Fig. 5:**
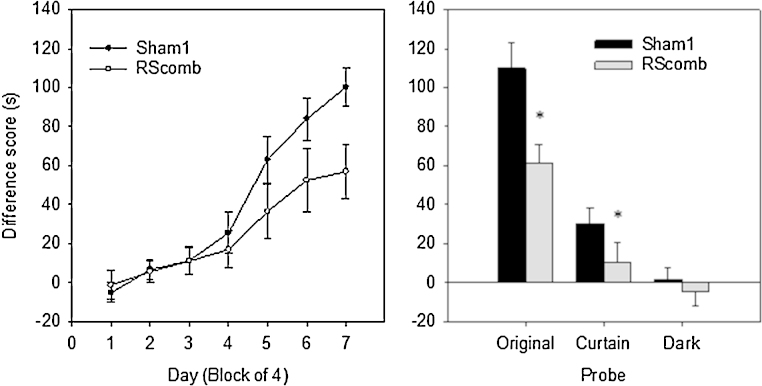
Experiment 1—Perspective task (Cohort 1). Left: Graph showing acquisition of the Perspective discrimination task by Sham1 and combined granular and dysgranular retrosplenial (RScomb) lesion animals. Rats discriminated between two spatial views, each with their own associated direction of travel. Performance is shown as the cumulative difference in dig latencies between incorrect (‘no-go’) and correct (‘go’) trials. Error bars show the standard error of the mean. Right: Histograms showing the performance of the Sham1 group and RScomb groups on the probe trials administered following acquisition of the perspective discrimination task. ‘Original’ refers to performance on the final day of training. When visual cues were occluded (‘Curtain’) both groups’ performance fell. Neither group performed above chance in the dark with the curtain drawn (‘Dark’). Error bars show the standard error of the mean. Asterisk (*) shows group difference *p* < 0.05.

During acquisition (Fig. 5 Left) the latency difference for go and no/go trials increased, as shown by a main effect of block (*F*(6, 114) = 53.4, *p* < 0.001). Although there was no overall difference between the lesion and control groups (*F*(1, 19) = 2.00, *p* > 0.05), there was a significant block by lesion interaction (*F*(6, 114) = 2.58, *p* < 0.05) (see Fig. 5 Left). Analysis of the simple main effects showed that although the two groups did not differ in the first 4 blocks (all *F* < 1), the Sham2 group performed significantly better than the RScomb rats in blocks 6 and 7 (of the two blocks, minimum *F*(1, 6.6) = 7.40, *p* < 0.05).

Both the Sham1 and RScomb groups appeared to use the distal room cues to perform the task as their latency difference scores dropped significantly compared to the final day of acquisition when the curtain was drawn around the testing arena (*F*(1, 19) = 54.6, *p* < 0.001; Fig. 5 Right). While the lack of significant trial type by lesion interaction *F*(1, 19) = 2.58, *p* > 0.05) might indicate that both groups were similarly affected by the Curtain probe, this result is difficult to interpret as the performance of the two groups started from different levels. During the Curtain probe the Sham1 animals were still able to discriminate the two directions (one sample test, *t*(11) = 4.05, *p* < 0.01), while the lesion animals’ performance fell to chance (*t* < 1). This difference in performance was reflected by a significant group difference on this probe (*F*(1, 19) = 11.6, *p* < 0.01; see Fig. 5 Right).

When further visual cues were removed by running the experiment in the dark, neither group could perform above chance (one sample *t* test, both *t* < 1; Fig. 5 Right). Although there was a significant decrease in performance compared to the Curtain probe (*F*(1, 19) = 10.7, *p* < 0.01), there was no trial type by lesion interaction (*F*(1, 19) = 1.04, *p* > 0.05). In the dark, both groups of animals dug equally during go and no-go trials (Sham1 mean latency to dig go trials = 6.69, no-go trials = 6.89; RScomb mean latency to dig go trials = 4.87, no-go trials = 4.32).

Between each of the two probe sessions the task was run again in the same way as during the acquisition phase, to prevent extinction. No difference was found between the final day of acquisition training and the two inter-probe days (*F*(2, 38) = 2.09, *p* > 0.05), and no lesion by trial interaction (*F* < 1). However, there was still a main effect of lesion (*F*(1, 19) = 10.3, *p* < 0.01), due to the poorer performance of the RScomb group compared to the Sham1 group.

### 3.3 Experiment 2—digging cup discrimination (non-spatial go/no-go)

#### 3.3.1 Combined retrosplenial lesions

**Fig. 6:**
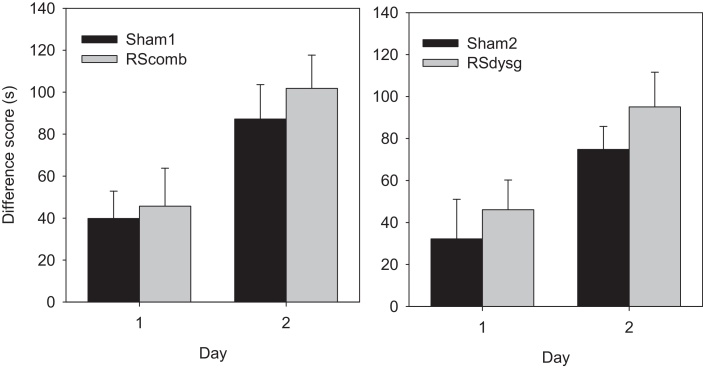
Experiment 2—Digging cup discrimination (Cohort 1, left, and Cohort 2, right). The histograms show the mean cumulative latency difference scores between incorrect (‘no-go’) and correct (‘go’) trials for the two sessions of testing ± standard error of the mean. Neither dysgranular retrosplenial (RSdysg) nor combined granular and dysgranular retrosplenial (RScomb) lesions affected performance on this non-spatial go/no-go discrimination. Rats began to acquire the discrimination within the first session, leaving overall performance above chance.

Analyses based on the latency difference scores summed across each session showed that by the second test day both the RScomb and Sham1 groups performed above chance levels (one sample *t* tests, RScomb; *t*(8) = 4.61, *p* < 0.01; Sham1; *t*(11) = 5.52, *p* < 0.001; see Fig. 6 Left). There was no lesion effect (*F* < 1) with respect to the latency difference scores for the no-go and go trials, although there was a main effect of day (*F*(1, 19) = 16.2, *p* < 0.001) demonstrating an improvement in performance with training. There was no day by lesion interaction (*F* < 1).

#### 3.3.2 Retrosplenial dysgranular lesions

Analyses based on the latency difference scores showed again that by the second test day both groups performed above chance (one sample test, RSdysg; *t*(7) = 9.38, *p* < 0.001; Sham2: *t*(9) = 4.52, *p* < 0.001). There was no significant difference in these scores between the two groups (*F*(1, 16) = 2.52, *p* > 0.05) and no day by lesion interaction (*F* < 1). The main effect of day (*F*(1, 16) = 20.6, *p* < 0.001) demonstrated that the rats improved with training (see Fig. 6 Right).

### 3.4 Experiment 3—place discrimination (‘location’ task)

#### 3.4.1 Combined retrosplenial lesions

**Fig. 7:**
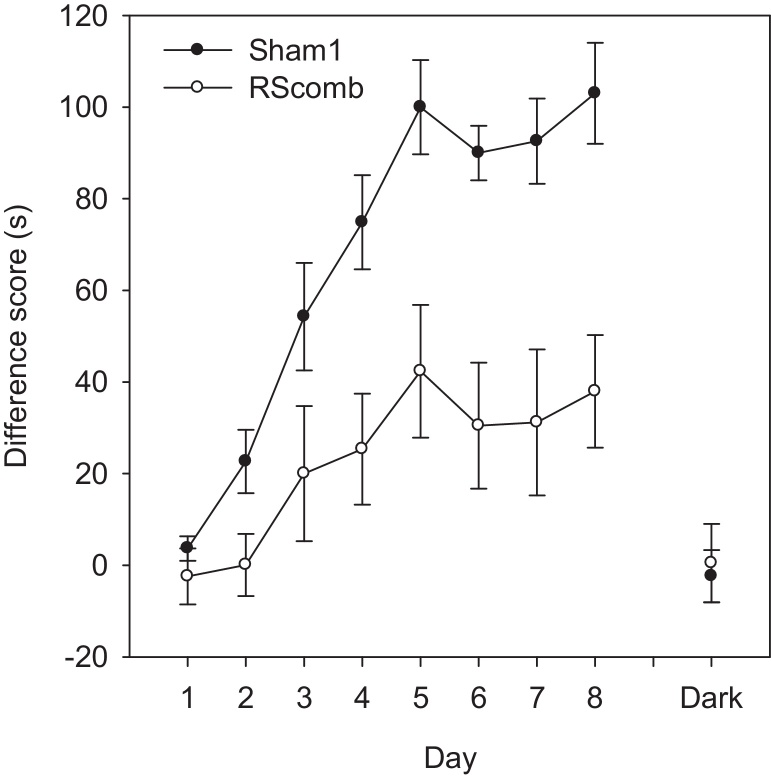
Experiment 3—Location task (Cohort 1). Graph showing acquisition of the Location task, where animals were rewarded for digging if the arena was in one corner of the room, but not in the opposite corner. Performance was measured as the cumulative difference between digging latencies on incorrect (‘no-go’) and correct (‘go’) trials. A higher difference score indicates superior discrimination. Although both groups performed significantly above chance level by the end of training, animals with combined lesions of the granular and dysgranular retrosplenial cortex (RScomb) showed retarded acquisition of this perspective discrimination task. Neither group was able to perform the same discrimination successfully in the dark. Error bars show the standard error of the mean.

For each animal, the latency difference between no-go and go trials was summed over the 16 trials in each session. Based on these latency difference scores (Fig. 7), the Sham1 group performed significantly better than the lesioned animals, as shown by a main effect of lesion (*F*(1, 19) = 11.3, *p* < 0.01) as well as a significant day × lesion interaction (*F*(7, 133) = 2.42, *p* < 0.05)). While there was also a main effect of day (*F*(7, 133) = 21.2, *p* < 0.001), indicating that the animals’ performance on the place discrimination task improved with training, the lesion effect and interaction show that this improvement predominantly reflected the scores of the Sham1 rats.

When the animals were tested in the dark, performance markedly decreased (Fig. 7). Indeed, neither group performed above chance during this Dark probe (both *t* < 1) nor did the two groups differ from each other (*t* < 1). These results reveal that the rats had relied on visual cues to solve the task. During dark trials, both groups of rats dug equally quickly in both the go and no-go trials (Sham1 mean latency to dig go trials = 6.18 s, no-go trials = 6.51 s; RScomb mean latency to dig go trials = 4.83 s, no-go trials = 4.94 s).

#### 3.4.2 Retrosplenial dysgranular lesions

**Fig. 8:**
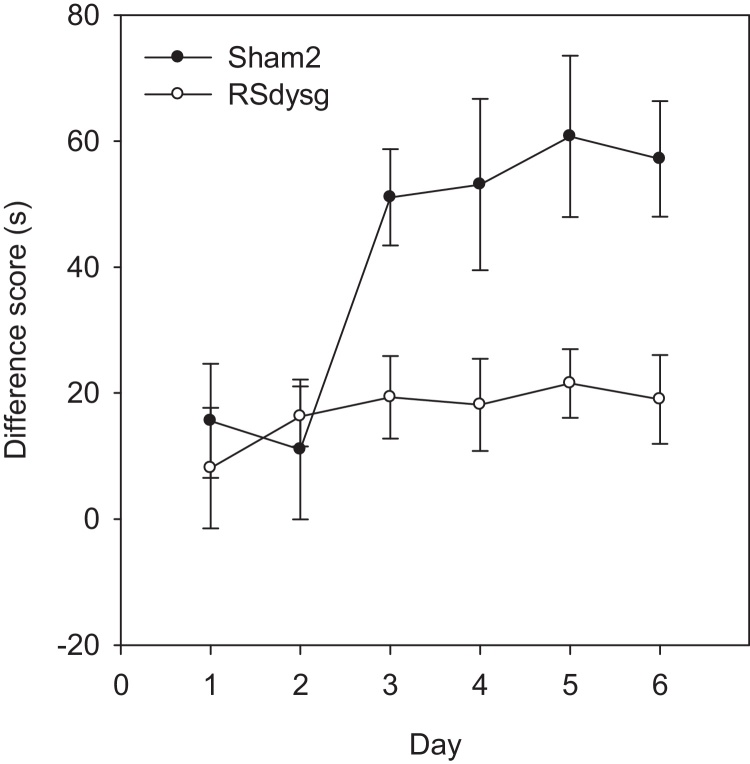
Experiment 3—Location task (Cohort 2). Graph showing acquisition of Location task, where rats were rewarded for digging in one corner of the room but not in the opposite corner. Performance is measured as the cumulative digging latency difference between incorrect (‘no-go’) and correct (‘go’) trials. A higher difference score indicates superior discrimination. Although both groups performed significantly above chance levels by the end of training, dysgranular retrosplenial lesioned animals (RSdysg) acquired this location discrimination task more slowly than the Sham2 animals. Error bars show the standard error of the mean.

The RSdysg lesion group was significantly poorer than their control group at discriminating between the two locations, as demonstrated by a main effect of group (*F*(1, 16) = 6.28, *p* < 0.05) when comparing the latency difference scores. There was, however, a significant main effect of day (*F*(5, 80) = 6.26, *p* < 0.001), showing that overall performance improved with training (Fig. 8), though there was no day by lesion interaction (*F*(5, 80) = 2.13, *p* = 0.071).

## 4 Discussion

The present study examined the effects of extensive retrosplenial cortex lesions (RScomb) involving both areas 29 and 30, as well as the effects of more selective lesions (RSdsyg) largely confined to the dysgranular cortex (area 30). As explained, only the findings from the rats with combined area 29 and 30 lesions are described for Experiment 1 (‘Perspective’ task). This task taxed the ability to discriminate a particular room view, along with its associated direction of travel, from the opposite (180^0^ rotated) room view and its associated direction of travel (Fig. 1). The RScomb rats were significantly impaired at learning this Perspective task. In the second spatial discrimination (Experiment 3, ‘Location’ task) both sets of lesions (i.e., RSdysg and RScomb) impaired a place discrimination task that required the rats to determine their location in a room using distal visual cues. Unlike the first experiment, no particular direction of travel was associated with reward as the same heading directions were used in both test locations. As Experiments 1 and 3 involved spatial go/no-go discriminations, Experiment 2 consisted of a non-spatial go/no-go discrimination. Neither cohort of rats with retrosplenial cortex lesions was impaired on this digging discrimination.

Evidence that the Perspective task (Experiment 1) normally required the use of distal visual stimuli came from the fall in performance when a curtain was closed around the test arena. Indeed, neither the control nor the lesion group performed above chance when subsequently tested in the dark. Consequently, these results support other findings implicating the retrosplenial cortex in integrating visual with spatial information [30,31]. The importance of this region for the effective use of distal visual cues is reflected in the bias following retrosplenial cortex lesions to use intra-maze cues to solve spatial tasks [18,32,33]. The Sham1 animals did, however, perform above chance when tested in the light with the curtain closed around the test arena (Fig. 5), suggesting that normal rats could use visual room cues that the rats with retrosplenial damage did not exploit. Examples of remaining visual cues in the Curtain probe include the placement of task-related equipment on the ceiling and the geometric features of the room, as the curtains did not obscure the room's shape. Rats can use relative length cues for location learning [34,35], an ability disrupted both by hippocampal and anterior thalamic lesions [36,37]. It is, therefore, possible that this same geometric ability is lost after retrosplenial lesions.

A key issue is the extent to which Experiment 1 was solved by discriminating the different directions of travel. It is known that head direction cells, which provide compass-like information, can be maintained by both distal visual cues and interoceptive cues [38], though the results of the probe tests highlight the importance of visual inputs for the present task. It is also known that head direction cells are found across both granular and dysgranular retrosplenial cortex [11,39]. Other relevant evidence comes from the finding that retrosplenial lesions can impair an alternation task in a cross-maze designed to tax direction information [16]. The report that retrosplenial lesions disrupt the stability of anterior thalamic head direction cells in the light, but have less effect on self-movement cues in the dark [40], would predict that the disruptive effects of retrosplenial lesions might be particularly evident for tasks dependent on visual feedback, as was the case in the present study. It was also shown in an additional probe test given only to Cohort 2 (data not presented) that in Experiment 1 the rats were not discriminating the absolute position of the digging cup within the room, which varied slightly according to trial type (Fig. 1). The parsimonious conclusion is, therefore, that retrosplenial cortex lesions impaired the Perspective task via their impact on head direction information. It was for this reason that Experiment 3 provided a contrasting spatial problem in which heading direction information was less of a discriminatory cue.

The demands of the Location task (Experiment 3) appear more complex than those of Experiment 1. Direction of travel within the test room is of less help as both directions of travel within the room are equally associated with reward and non-reward (Fig. 2). Furthermore, the value of individual, distal room cues may also be more ambiguous than in Experiment 1 as both the rewarded and non-rewarded locations will be associated with overlapping common cues, albeit from different distances. Despite appearing more complex, it is striking that the Sham1 rats took appreciably less time to master Experiment 3 than Experiment 1 (Figs. 5 and 7). One contributing factor is likely to be positive transfer from Experiment 1 for some task aspects. Another factor is that learning in which place food is present, irrespective of direction travelled, is far more ethologically appropriate than learning in which direction of travel is food associated, when location is irrelevant. Finally, the locations to be discriminated in Experiment 3 were adjacent to walls while the arena for Experiment 1 was placed in the centre of the test room. In pilot studies with normal rats it was found that location go/no-go discriminations become appreciably more difficult when the rat is placed further and further away from the side walls. Presumably, cues on the nearest walls dominate performance, so aiding the discrimination in Experiment 3.

The finding that extensive retrosplenial lesions (RScomb) disrupt the ability to discriminate locations may seem unsurprising given the deficits on tasks such as the Morris water maze [7,17,41]. In a standard Morris water maze, rats are presumed to learn a platform location with reference to multiple room cues, e.g., furniture, windows, wall hangings, and lights, using a cognitive map that can be applied in a flexible manner [42,43,44; but see 45]. Additional demands in the Morris water maze include the ability to navigate effectively to the platform location and to avoid repeat visits to any location without the platform. The present task not only removes the navigational requirement but also reduces the need for the animal to monitor where it has travelled within that trial. Despite these apparent simplifications, retrosplenial cortex lesions still markedly impaired acquisition. The finding that the complete retrosplenial lesions did not completely stop learning can be related back to the existence of multiple brain sites supporting spatial learning [1].

A feature of the Location task (Experiment 3) was the clear deficit shown by the rats with lesions largely confined to the dysgranular retrosplenial cortex (Fig. 8). This deficit is presumably linked to the visual demands of the problem [32]. It is, therefore, notable that anatomical studies reveal how visual information first reaching the retrosplenial cortex preferentially targets the dysgranular region [5,22,46]. This same distinction is supported by immediate-early gene imaging experiments that reveal contrasting activity measures in the dysgranular and granular retrosplenial cortices associated with spatial learning in the light and dark, respectively [47]. The present findings, therefore, support the notion that area 30 is a critical route for the engagement of both areas 29 and 30 by distal visual stimuli. At the same time, previous studies into the impact of lesions targeted at just the granular retrosplenial cortex (area 29) help to emphasize how these two areas often support the same classes of spatial problem [33].

In summary, the current experiments highlight the importance of the rat retrosplenial cortex for the use of distal visuo-spatial information. One description that would fit the pattern of results from selective retrosplenial cortex lesion studies [32,33,48] is that the two retrosplenial regions (areas 29 and 30) work closely together to combine and integrate exteroceptive (e.g., visual stimuli, mainly area 30) and interoceptive (e.g., proprioceptive stimuli, mainly area 29) cues to resolve spatial problems that involve the integration of different information types, including the resolution of multiple viewpoints from the same location (‘stimulus translation’). The finding that selective lesions of area 29 (granular cortex) can disrupt visuo-spatial tasks, e.g., the Morris water maze [33,48], would seem to underline the strong inter-dependence between retrosplenial subareas, which is effected by their many interconnections [49]. This interactive process could then be a key element in allowing the animal to use different spatial frames of reference to navigate effectively*.* A final point concerns the apparent simplicity of the present tasks yet their sensitivity to retrosplenial cortex lesions. In contrast, other spatial tests run in ‘dry’ mazes such as T-maze alternation or radial-arm maze nonmatching are often surprisingly insensitive to retrosplenial cortex damage when run in a standard manner [16,18,28,32], presumably reflecting the variety of solutions available to the animals. For this reason, the further development of spatial go/no-go tasks offers a route to understand better the specific contributions of retrosplenial cortex to spatial learning.

## References

[1] Mizumori S.J., Ragozzino K.E., Cooper B.G., Leutgeb S. (1999). Hippocampal representational organization and spatial context. Hippocampus.

[2] Aggleton J.P., Wright N.F., Vann S.D., Saunders R.C. (2012). Medial temporal lobe projections to the retrosplenial cortex of the macaque monkey. Hippocampus.

[3] Morris R., Petrides M., Pandya D.N. (1999). Architecture and connections of retrosplenial area 30 in the rhesus monkey (Macaca mulatta). Eur J Neurosci.

[4] Van Groen T., Wyss J.M. (2003). Connections of the retrosplenial granular b cortex in the rat. J Comp Neurol.

[5] Van Groen T., Wyss J.M. (1992). Connections of the retrosplenial dysgranular cortex in the rat. J Comp Neurol.

[6] Van Groen T., Wyss J.M. (1990). Extrinsic projections from area CA1 of the rat hippocampus: olfactory, cortical, subcortical, and bilateral hippocampal formation projections. J Comp Neurol.

[7] Sutherland R.J., Hoesing J.M. (1993). Posterior cingulate cortex and spatial memory: a microlimnology analysis. Neurobiology of Cingulate Cortex and Limbic Thalamus.

[8] Vogt B.A., Pandya D.N. (1987). Cingulate cortex of the rhesus monkey: II. Cortical afferents. J Comp Neurol.

[9] Vogt B.A., Pandya D.N., Rosene D.L. (1987). Cingulate cortex of the rhesus monkey: I. Cytoarchitecture and thalamic afferents. J Comp Neurol.

[10] Chen L.L., Lin L.H., Green E.J., Barnes C.A., McNaughton B.L. (1994). Head-direction cells in the rat posterior cortex. I. Anatomical distribution and behavioral modulation. Exp Brain Res.

[11] Cho J., Sharp P.E. (2001). Head direction, place, and movement correlates for cells in the rat retrosplenial cortex. Behav Neurosci.

[12] Maguire E.A. (2001). The retrosplenial contribution to human navigation: a review of lesion and neuroimaging findings. Scand J Psychol.

[13] Takahashi N., Kawamura M., Shiota J., Kasahata N., Hirayama K. (1997). Pure topographic disorientation due to right retrosplenial lesion. Neurology.

[14] Vann S.D., Aggleton J.P., Maguire E.A. (2009). What does the retrosplenial cortex do?. Nat Rev Neurosci.

[15] Haijima A., Ichitani Y. (2008). Anterograde and retrograde amnesia of place discrimination in retrosplenial cortex and hippocampal lesioned rats. Learn Mem.

[16] Pothuizen H.H.J., Aggleton J.P., Vann S.D. (2008). Do rats with retrosplenial cortex lesions lack direction?. Eur J Neurosci.

[17] Vann S.D., Aggleton J.P. (2002). Extensive cytotoxic lesions of the rat retrosplenial cortex reveal consistent deficits on tasks that tax allocentric spatial memory. Behav Neurosci.

[18] Vann S.D., Aggleton J.P. (2004). Testing the importance of the retrosplenial guidance system: effects of different sized retrosplenial cortex lesions on heading direction and spatial working memory. Behav Brain Res.

[19] Whishaw I.Q., Maaswinkel H., Gonzalez C.L., Kolb B. (2001). Deficits in allothetic and idiothetic spatial behavior in rats with posterior cingulate cortex lesions. Behav Brain Res.

[20] Cooper B.G., Mizumori S.J. (1999). Retrosplenial cortex inactivation selectively impairs navigation in darkness. Neuroreport.

[21] Zheng Y., Pearce J.M., Vann S.D., Good M., Jenkins T.A., Smith P.F. (2003). Using idiothetic cues to swim a path with a fixed trajectory and distance: necessary involvement of the hippocampus, but not the retrosplenial cortex. Behav Neurosci.

[22] Mizumori S.J., Cooper B.G., Leutgeb S., Pratt W.E. (2000). A neural systems analysis of adaptive navigation. Mol Neurobiol.

[23] Burgess N., Becker S., King J.A., O’Keefe J. (2001). Memory for events and their spatial context: models and experiments. Philos Trans R Soc Lond B Biol Sci.

[24] Byrne P., Becker S., Burgess N. (2007). Remembering the past and imagining the future: a neural model of spatial memory and imagery. Psychol Rev.

[25] Epstein R.A., Parker W.E., Feiler A.M. (2007). Where am I now? Distinct roles for parahippocampal and retrosplenial cortices in place recognition. J Neurosci.

[26] Lambrey S., Doeller C., Berthoz A., Burgess N. (2012). Imagining being somewhere else: neural basis of changing perspective in space. Cereb Cortex.

[27] Gonzalez C.L., Whishaw I., Kolb B. (2003). Complete sparing of spatial learning following posterior and posterior plus anterior cingulate cortex lesions at 10 days of age in the rat. Neuroscience.

[28] Neave N., Lloyd S., Sahgal A., Aggleton J.P. (1994). Lack of effect of lesions in the anterior cingulate cortex and retrosplenial cortex on certain tests of spatial memory in the rat. Behav Brain Res.

[29] Vann S.D., Wilton L.A.K., Muir J.L., Aggleton J.P. (2003). Testing the importance of the caudal retrosplenial cortex for spatial memory in rats. Behav Brain Res.

[30] Cooper B.G., Mizumori S.J. (2001). Temporary inactivation of the retrosplenial cortex causes a transient reorganization of spatial coding in the hippocampus. J Neurosci.

[31] Cooper B.G., Manka T.F., Mizumori S.J. (2001). Finding your way in the dark: the retrosplenial cortex contributes to spatial memory and navigation without visual cues. Behav Neurosci.

[32] Vann S.D., Aggleton J.P. (2005). Selective dysgranular retrosplenial cortex lesions in rats disrupt allocentric performance of the radial-arm maze task. Behav Neurosci.

[33] Pothuizen H.H.J., Davies M., Aggleton J.P., Vann S.D. (2010). Effects of selective granular retrosplenial cortex lesions on spatial working memory in rats. Behav Brain Res.

[34] Cheng K. (1986). A purely geometric module in the rat's spatial representation. Cognition.

[35] McGregor A., Hayward A.J., Pearce J.M., Good M.A. (2004). Hippocampal lesions disrupt navigation based on the shape of the environment. Behav Neurosci.

[36] Jones P.M., Peace J.M., Davies V.J., Good M.A., McGregor A. (2007). Impaired processing of local geometric features during navigation in a water maze following hippocampal lesions in rats. Behav Neurosci.

[37] Aggleton J.P., Poirier G.L., Aggleton H.S., Vann S.D., Pearce J.M. (2009). Lesions of the fornix and anterior thalamic nuclei dissociate different aspects of hippocampal-dependent spatial learning: Implications for the neural basis of scene learning. Behav Neurosci.

[38] Taube J.S. (2007). The head direction signal: origins and sensory motor integration. Annu Rev Neurosci.

[39] Chen L.L., Lin L.H., Barnes C.A., McNaughton B.L. (1994). Head-direction cells in the rat posterior cortex. II. Contributions of visual and ideothetic information to the directional firing. Exp Brain Res.

[40] Clark B.J., Bassett J.P., Wang S.S., Taube J.S. (2010). Impaired head direction cell representation in the anterodorsal thalamus after lesions of the retrosplenial cortex. J Neurosci.

[41] Cain D.P., Humpartzoomian R., Boon F. (2006). Retrosplenial cortex lesions impair water maze strategies learning or spatial place learning depending on prior experience of the rat. Behav Brain Res.

[42] Tolman E.C. (1948). Cognitive maps in rats and men?. Psychol Rev.

[43] O’Keefe J., Nadel L. (1978). The Hippocampus as a Cognitive Map.

[44] Morris R.G.M. (1981). Spatial localisation does not depend on the presence of local cues. Learn Motivat.

[45] Hamilton D.A., Akers K.G., Johnson T.E., Rice J.P., Candelaria F.T., Redhead E.S. (2009). Evidence for a shift from place navigation to directional responding in one variant of the Morris water task. J Exp Psychol Anim Behav Process.

[46] Vogt B.A., Miller M.W. (1983). Cortical connections between rat cingulate cortex and visual, motor, and postsubicular cortices. J Comp Neurol.

[47] Pothuizen H.H.J., Davies M., Albasser M.M., Aggleton J.P., Vann S.D. (2009). Granular and dysgranular retrosplenial cortices provide qualitatively different contributions to spatial working memory: evidence from immediate-early gene imaging in rats. Eur J Neurosci.

[48] Van Groen T., Kadish I., Wyss J.M. (2004). Retrosplenial cortex lesions of area Rgb (but not Rga) impair spatial learning and memory in the rat. Behav Brain Res.

[49] Shibata H., Honda Y., Sasaki H., Naito J. (2009). Organization of intrinsic connections of the retrosplenial cortex in the rat. Anat Sci Int.

[50] Paxinos G., Watson C. (2006). The Rat Brain in Stereotaxic Coordinates.

